# Resolution of Symptomatic Intracranial Hypertension Following Resection of Tentorial Meningioma Compressing the Dominant Transverse Sinus

**DOI:** 10.1055/a-2650-6754

**Published:** 2025-07-22

**Authors:** Samuel B. Tomlinson, Redi Rahmani, Rashad Jabarkheel, Adam M. Kruszewski, Daniel Yoshor, Visish M. Srinivasan

**Affiliations:** 1 Department of Neurosurgery, Perelman School of Medicine, University of Pennsylvania, Philadelphia, Pennsylvania, United States; 2 Division of Neuro-Ophthalmology, Department of Neurology, Perelman School of Medicine, University of Pennsylvania, Philadelphia, Pennsylvania, United States

**Keywords:** tentorial meningioma, intracranial hypertension, venous manometry, venous outflow obstruction, elevated intracranial pressure, transverse sinus

## Abstract

**Background:**

Symptomatic intracranial hypertension is a rare presentation of meningiomas associated with compression and/or invasion of the dural venous sinuses. Establishing a clear link between tumor-induced venous outflow obstruction and elevated intracranial pressure is essential to determine the appropriate management strategy.

**Case Description:**

A 59-year-old female presented with headaches, imbalance, pulsatile tinnitus, and horizontal binocular diplopia secondary to bilateral abducens nerve dysfunction in the setting of a small tentorial meningioma compressing the dominant right transverse sinus. Venous manometry demonstrated elevated sinus pressures and a large pressure gradient across the lesion. Microsurgical resection improved the caliber of the transverse sinus and normalized intracranial pressures without the need for permanent venous stent placement.

**Conclusion:**

Tentorial meningiomas infrequently result in venous outflow obstruction and symptomatic intracranial hypertension. Thorough workup including diagnostic angiography, venous manometry, and temporary stenting can be used to confirm the diagnosis. Surgical resection with or without permanent stent placement can restore venous drainage and alleviate debilitating symptoms.

## Introduction

Symptomatic intracranial hypertension caused by compression and/or invasion of the dural venous sinuses is an uncommon presentation of intracranial meningiomas. 1 Tentorial meningiomas exerting mass effect on the transverse–sigmoid complex can result in venous outflow obstruction and elevated intracranial pressure (ICP). 2 For small tentorial meningiomas, establishing a causal link between sinus compression, elevated ICPs, and associated symptomatology is critical for rationalizing surgical intervention over surveillance. In this report, we describe the case of a 59-year-old female who presented with symptomatic intracranial hypertension secondary to extrinsic compression of the dominant transverse sinus by a small tentorial meningioma. Venous manometry revealed a large pressure gradient across the lesion, and expansion of a temporary venous stent-retriever improved sinus caliber at the site of focal stenosis. Microsurgical resection restored normal sinus pressures and resolved the patient's debilitating symptoms without the need for permanent stenting.

## Case Presentation

**Video 1** Resolution of symptomatic intracranial hypertension following resection of tentorial meningioma compressing the dominant transverse sinus.


A 59-year-old female was evaluated for progressive horizontal binocular diplopia, imbalance, occipital headaches, and pulsatile tinnitus. Diplopia was present for about 10 years and had been managed with prism glasses. Over several months prior to presentation, her diplopia rapidly worsened and she developed new progressive positional headaches exacerbated by bending over and lying flat. Neuro-ophthalmologic examination revealed incomitant esotropia that increased in right and left gazes, as well as trace abduction deficits in each eye suggestive of bilateral abducens nerve dysfunction. Fundoscopic examination showed no frank papilledema. Visual acuity, color vision, and visual field testing were normal. Additional workup included negative serum acetylcholine receptor antibody testing, normal single fiber orbicularis oculi muscle electromyography, and normal repetitive nerve stimulation studies. Intracranial hypertension symptoms did not improve after several weeks of acetazolamide therapy.

**Fig. 1:**
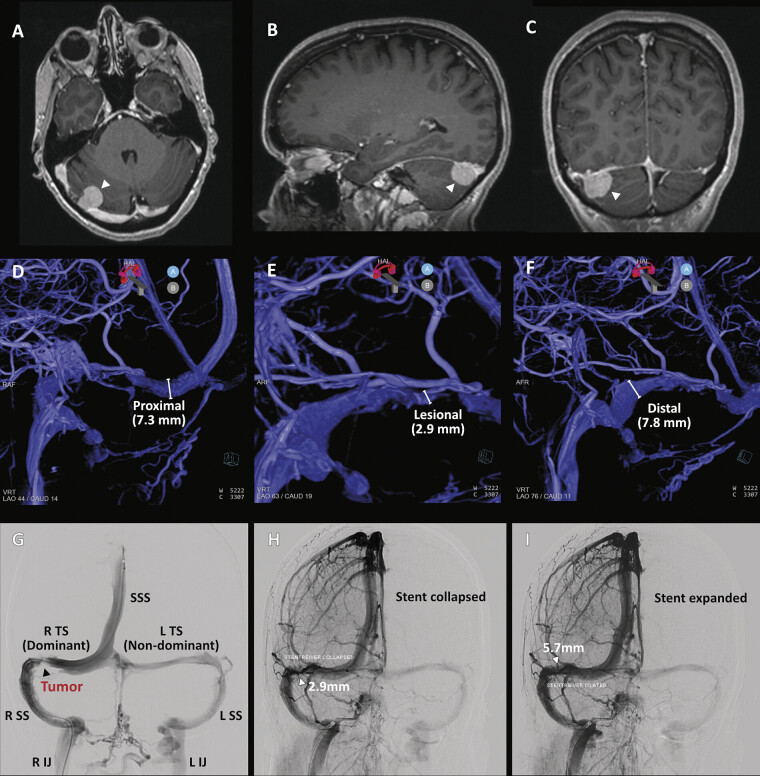
Small tentorial meningioma compressing the dominant right transverse sinus. ( **A–C** ) Preoperative axial ( **A** ), sagittal ( **B** ), and coronal ( **C** ) T1-weighted postcontrast MRI images demonstrating small tentorial meningioma (1.7 × 1.7 × 1.4 cm) compressing the dominant right transverse sinus at the proximal transverse–sigmoid junction resulting in subtotal obstruction. ( **D** **–F** ) Venous-phase angiography showing focally diminished transverse sinus at the site of extrinsic tumor compression. ( **G** ) Venous angiogram (superior sagittal sinus) revealing focal tumor-induced stenosis at the proximal right transverse–sigmoid junction (arrowhead). ( **H, I** ) Expansion of a temporary stent-retriever spanning the segment of focal stenosis demonstrated restoration of sinus caliber (2.9 mm versus 5.7 mm). IJ, internal jugular; SS, sigmoid sinus; SSS, superior sagittal sinus; TS, transverse sinus.

MRI of the head with and without intravenous contrast demonstrated bilateral optic nerve sheath tortuosity and dilation, unobstructed cerebrospinal fluid (CSF) pathways without ventriculomegaly, and a small tentorial meningioma (1.7 × 1.7 × 1.4 cm) compressing the dominant right transverse sinus at the proximal transverse–sigmoid junction associated with subtotal transverse sinus occlusion without clear intraluminal invasion ( Fig. 1A – C ).

To investigate a potential link between sinus outflow obstruction and intracranial hypertension, the patient underwent lumbar puncture (LP), angiography, venous manometry, and temporary stent-retriever placement. LP opening pressure (OP) was elevated (40–45 cm H _2_ O). Venous-phase 3D rotational angiography demonstrated a dominant right transverse sinus, focal stenosis at the site of meningioma compression, and engorgement of the cavernous sinus and occipital condylar plexus ( Fig. 1D – G ). Venous manometry revealed a large pressure gradient across the lesion at the proximal aspect of the transverse–sigmoid junction ( Table 1 ). Expansion of a temporary stent-retriever (Tigertriever 21, Rapid Medical, Yokneam, Israel) resulted in improved vessel caliber (2.9 to 5.7 mm), with reversion to original diameter after relaxation ( Fig. 1H , I ).

**Table 1:** Venous manometry measurements before and after resection of tentorial meningioma compressing the dominant right transverse sinus

Location	Pre-resection (cm H _2_ O)	Post-resection (cm H _2_ O)	Difference (cm H _2_ O, %Δ)
SSS	30	7	−23 (−76.7%)
Torcula	25	9	−16 (−64.0%)
R TS (mid)	21	6	−15 (−71.4%)
R TS-SS junction (proximal)	23	6	−17 (−73.9%)
R TS-SS junction (lesional)	10	6	−4 (−40.0%)
R TS-SS junction (distal)	5	6	+1 (+20.0%)
R SS	5	8	+3 (+60.0%)
R IJ	5	6	+1 (+20.0%)

Abbreviations: IJ, internal jugular; SS, sigmoid sinus; SSS, superior sagittal sinus; TS, transverse sinus.

**Fig. 2:**
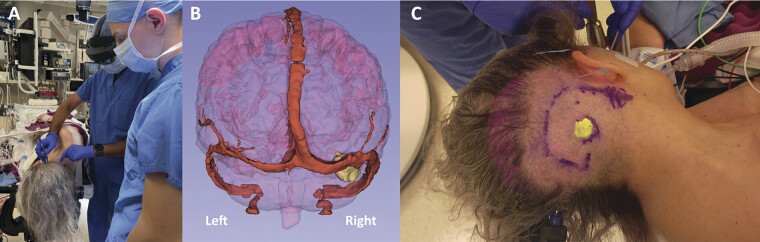
Surgical positioning and incision planning. An augmented reality (AR)-based neuronavigation system (Hoth Intelligence, Philadelphia, PA) was used to localize the tumor and plan the incision. ( **A** ) Surgeon visualizes the AR projection through the Microsoft HoloLens. ( **B** ) Rendering of the AR display (posterior view) showing the venous sinus anatomy (red) and tumor (yellow). ( **C** ) AR projection of the tumor (yellow) facilitated planning of skin incision.

After thorough discussion of treatment options including ventriculoperitoneal shunting and venous sinus stenting without tumor resection, the patient consented to undergo retrosigmoid craniotomy for tumor excision followed by intraoperative angiography and repeat manometry. The procedure was performed in a hybrid operating suite equipped with biplanar fluoroscopy. A lumbar drain was placed to facilitate CSF drainage throughout the case. Arterial and venous access was obtained via the right radial artery (5-Fr sheath) and antecubital vein (6-Fr sheath), respectively. The patient was positioned laterally with the head rotated to bring the right post-auricular region to the apex of the field. An inverted U-shaped incision was planned using a combination of Medtronic StealthStation and augmented reality-based neuronavigation (Hoth Intelligence, Philadelphia, PA) ( Fig. 2 ). A generous myocutaneous flap was reflected inferiorly to expose the right subocciput. A retrosigmoid craniotomy was fashioned to unroof the transverse–sigmoid junction. The C-shaped dural leaflet was flapped superiorly toward the transverse sinus.

**Fig. 3:**
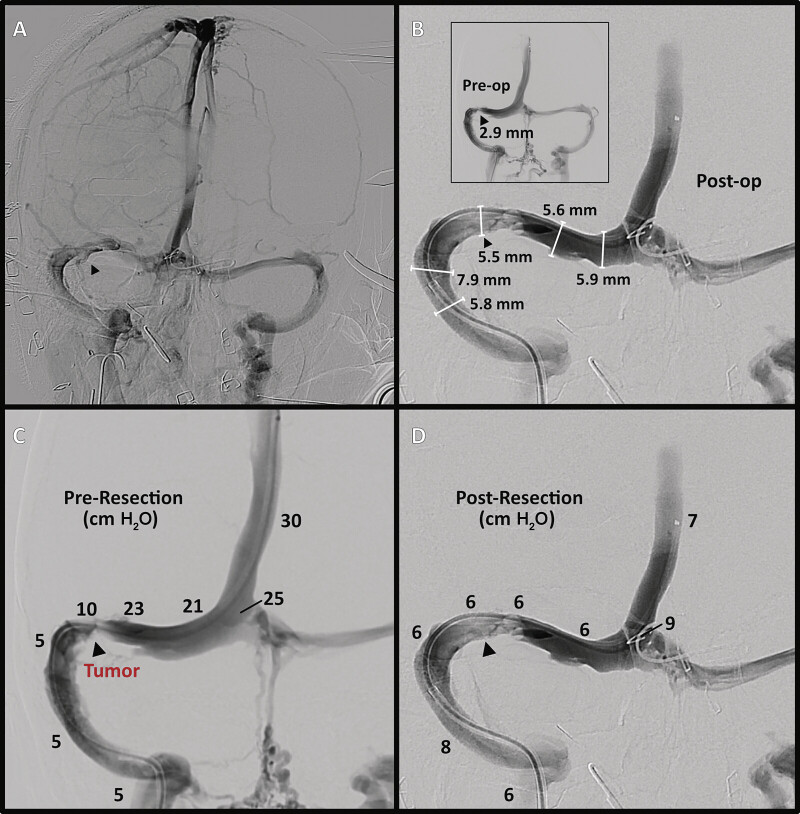
Restoration of sinus caliber and normalization of venous pressures following tumor resection. ( **A** ) Intraoperative diagnostic angiogram (venous phase, right internal carotid injection) revealing improved caliber of R transverse sinus at the site of previous focal compression (arrowhead). ( **B** ) Venous angiogram (superior sagittal sinus) further illustrating improved sinus diameter compared with pre-resection (inset). ( **C, D** ) Venous manometry measurements before ( **C** ) versus after ( **D** ) tumor resection demonstrating normalized sinus pressures and resolved pressure gradient across the transverse–sigmoid junction. Decreased collateral drainage through the midline venous plexus is also apparent.

Under the operating microscope ( Supplementary Video 1 ), the tentorial meningioma was identified and carefully dissected from the surrounding cerebellum. The tumor was internally debulked and de-vascularized at its base with care to preserve the patency of the transverse sinus. A small focus of possible microscopic tumor infiltration into the sinus was explored, but ultimately, entry into the sinus was deemed unnecessary. Intraoperative venous angiography after tumor resection demonstrated sinus patency, improved venous drainage, and restoration of normal sinus diameter ( Fig. 3A , B ). Manometry revealed normalization of venous sinus pressures ( Table 1 ; Fig. 3C , D ), obviating the need for permanent stenting. After watertight dural closure, the bone flap was replaced, the incision was closed, and the lumbar drain was removed.

**Fig. 4:**
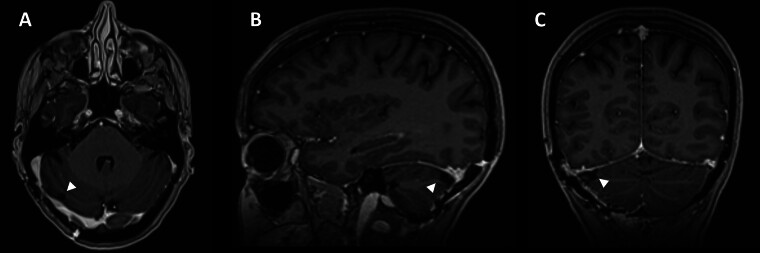
Complete resection of enhancing tumor. Postoperative axial ( **A** ), sagittal ( **B** ), and coronal ( **C** ) T1-weighted postcontrast MRI images demonstrating complete resection of tentorial meningioma (arrowhead) with preserved sinus patency.

Postoperative recovery was uneventful. MRI of the head with and without contrast (postoperative day 1; POD1) demonstrated complete resection of enhancing tumor and preserved sinus patency ( Fig. 4 ). She was discharged home on POD2. She experienced gradual resolution of her debilitating headaches, balance issues, and pulsatile tinnitus. Acetazolamide was successfully weaned off. By 3-month follow-up her binocular diplopia significantly improved, esotropia improved, and ocular motility was normal bilaterally, all suggesting improved abducens nerve function. Repeat MRI at 3 months revealed continued sinus patency and no evidence of tumor recurrence. Pathology was consistent with WHO grade I meningioma.

The participants and any identifiable individuals consented to publication of his/her image. The patient consented to publication of his/her image. Institutional review was not required for this case presentation as per institutional policy.

## Discussion

Although meningiomas frequently associate with the dural venous sinuses, their indolent growth typically permits the gradual maturation of venous collaterals that compensate for obstructed or even occluded sinuses. Previous reports of tumor-induced venous outflow obstruction resulting in symptomatic intracranial hypertension are rare. 1 3 4 5 6 7 8 9 10 This case illustrates diagnostic and technical nuances involved in managing this unusual presentation. First, convincing evidence favoring the uncommon diagnosis of tumor-induced venous hypertension over more common entities (e.g., idiopathic intracranial hypertension, migraine, etc.) should be pursued. Cheyuo et al 6 were the first to use venous manometry to demonstrate a pressure gradient across a segment of the sigmoid sinus compressed externally by a meningioma. This adjunct proved essential in our case given the small size of the meningioma and the partial sinus patency demonstrated on preoperative imaging. Second, selection of the optimal treatment strategy must consider factors such as venous drainage pattern and degree of sinus invasion. Most patients exhibit asymmetric flow through the transverse–sigmoid system, and surgical manipulation of the dominant side is typically avoided, especially when the sinus is partially patent. 11 Recently, dural sinus stenting has been used to restore venous drainage in patients with tumor-induced outflow obstruction, 12 13 although studies with long-term follow-up demonstrate that patients often require repeat intervention to maintain stent patency. 14 In our case, a temporary stent-retriever was used to confirm that normal sinus caliber could be restored with stenting, but we ultimately opted for upfront surgical resection given the accessibility of the tumor, the extrinsic source of compression without clear intraluminal extension, and the patient's preference for resection. Finally, our case illustrates the utility of the hybrid operating suite, which would have facilitated permanent stenting had post-resection angiography revealed persistent obstruction and elevated pressures.

## Conclusion

Symptomatic intracranial hypertension is a rare presentation of meningiomas associated with compression and/or invasion of the dural venous sinuses. Adjudicating between definitive resection and less-invasive alternatives requires assessment of the severity of the venous outflow obstruction, its potential reversibility, and the anticipated risks of sinus injury from surgical manipulation. In well-selected patients, tumor resection to relieve sinus obstruction can normalize ICPs and resolve associated symptoms.
